# Nanocellulose in Drug Delivery and Antimicrobially Active Materials

**DOI:** 10.3390/polym12122825

**Published:** 2020-11-27

**Authors:** Kaja Kupnik, Mateja Primožič, Vanja Kokol, Maja Leitgeb

**Affiliations:** 1 Laboratory for Separation Processes and Product Design, Faculty of Chemistry and Chemical Engineering, University of Maribor, Smetanova ulica 17, SI-2000 Maribor, Slovenia; kaja.kupnik@um.si (K.K.); mateja.primozic@um.si (M.P.); 2 Faculty of Mechanical Engineering, University of Maribor, Smetanova ulica 17, SI-2000 Maribor, Slovenia; vanja.kokol@um.si; 3 Faculty of Medicine, University of Maribor, Taborska ulica 8, SI-2000 Maribor, Slovenia

**Keywords:** nanocellulose, drug delivery, antimicrobial activity

## Abstract

In recent years, nanocellulose (NC) has also attracted a great deal of attention in drug delivery systems due to its unique physical properties, specific surface area, low risk of cytotoxicity, and excellent biological properties. This review is focused on nanocellulose based systems acting as carriers to be used in drug or antimicrobial delivery by providing different but controlled and sustained release of drugs or antimicrobial agents, respectively, thus showing potential for different routes of applications and administration. Microorganisms are increasingly resistant to antibiotics, and because, generally, the used metal or metal oxide nanoparticles at some concentration have toxic effects, more research has focused on finding biocompatible antimicrobial agents that have been obtained from natural sources. Our review contains the latest research from the last five years that tested nanocellulose-based materials in the field of drug delivery and antimicrobial activity.

## 1. Introduction

**Figure 1:**
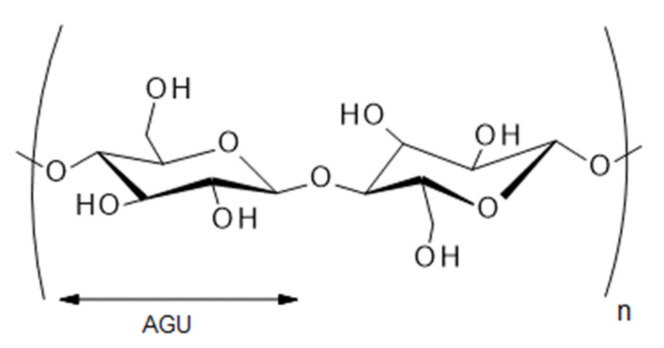
Structure of cellobiose that shows the repeated AGUs (summarized by [4]).

Cellulose is a major component of several plant tissues and, as such, the most abundant renewable polymer resource available [1,2]. It is a linear polymer of D-anhydroglucopyranose units (AGUs), connected by β-(1->4)-glycosidic bonds, where a repeat unit is a dimer of glucose, cellobiose (Figure 1) [3,4].

**Figure 2:**
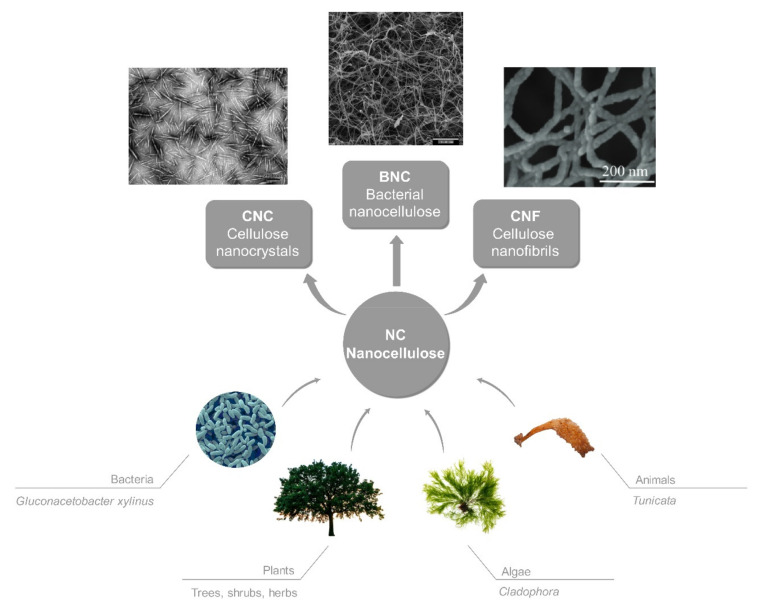
Different sources and types of nanocellulose with representative electron microscope images [8,9,10], reproduced by permission of The Royal Society of Chemistry [11,12,13,14].

Nanocellulose, in the form of fibrils (CNF) or crystals (CNC) [5,6], can be obtained from four main natural sources (Figure 2): bacteria, plants, algae, and animals [7].

While CNCs are prepared via acid hydrolysis [15,16,17], CNFs can be obtained by hydrolysis, oxidation and/or mechanical decomposition of biomass [18,19,20], or are produced by Gram-negative bacteria (mainly from *Gluconacetobacter xylinus* [21,22,23,24]) (Bacterial nanocellulose; BNC) [25], forming a highly viscous hydrogel when being suspended in water [26,27]. Among them, bacterial cellulose (BC) is the purest, contains no byproducts or contaminant molecules such as lignin, pectin, and hemicelluloses [28,29]. Its mechanical properties, ultrafine and highly porous network structure with high (over 95%) water-holding capacity, large surface area, and excellent biocompatibility [30] are its main advantages as compared to the plant-extracted NC, showing its extensive usage in the Cosmetic industry [31].

**Figure 3:**
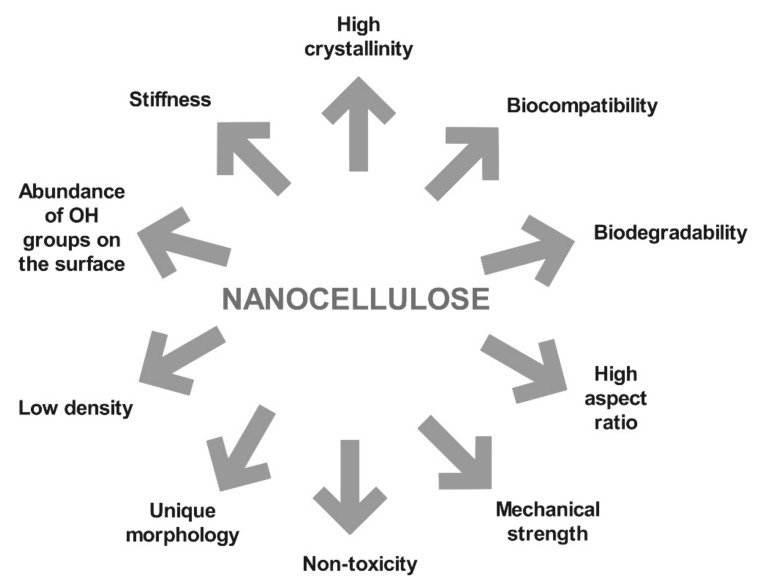
Unique properties allow the use of nanocellulose in many Biomedical applications [2].

High strength and stiffness combined with low weight [32] and low risk of cytotoxicity [6,33] (Figure 3), as well as the ability to be formed into larger two-dimensional (2D) and three-dimensional (3D) structures, including membranes, films, porous aerogels and foams [34,35,36,37,38], are other outstanding properties of NC. Its application has thus received increasing attention in many applications, besides the paper industry also in (bio) medicinal, pharmaceutical, cosmetics and the food industry [39,40,41,42,43]. Furthermore, the use and addition of nanocellulose as a drug carrier could, simultaneously, target the local drug delivery to reduce consumption and control the release of the incorporated drug [44]. Because of NCs high surface to volume ratio, a higher cellular binding and uptake is provided, which causes an increase in the effectiveness of such delivery systems [45]. The application of both external and internal delivery systems of nanocellulose-based drugs is possible [46].

An abundance of infectious diseases is occurring, and besides, antibiotic resistance is increasing. Therefore, it is necessary to search for new effective surface disinfection and alternative materials that strive for antimicrobial and other bioactive characteristics. The traumatic injuries and burn wound healing process can be extended if bacteria cause wound infection. Hence, particularly interesting are products derived from natural sources that would have antimicrobial properties and would inhibit the growth and reproduction of microorganisms [6,47].

It is known that most of the bacterial cell walls are negatively charged. Indeed, some compounds (quaternary ammonium compounds, molecules, or polymers) could interact electrostatically with the negatively charged bacterial cell wall. Therefore, it causes membrane disruption and subsequent posterior death [48,49]. Tissues based on cellulose are often impregnated with active substances (e.g., essential oils, plant extracts, etc.), which are bound inside the cellulose network. The abundance of hydroxyl groups in such a structure is perfect for active substances and water molecules to bond inside the hydrogen-bonded network, thus allowing these substances to bind and then transfer into the skin [50]. The hydroxyl groups on the surface and the relatively large specific surface area of NC also provide many options for its modification and functionalization. To obtain functional groups to the nanocellulose surface, and to use these compounds as precursors for further applications, covalent modifications (e.g., oxidation, esterification, etherification, polymerization, etc.) are usually used, as well as non-covalent binding of the functional molecules [51,52,53,54].

Natural polymers such as starch, gelatin, chitosan, collagen, and cellulose, have enormous proven potential in the Biomedicine field in recent years, with many studies [6,55]. In Biomedicine, biocompatibility with no toxic effect, immunological rejection and/or physiological reaction [56,57,58,59] is one of the most important features [60,61,62,63]. For healing of wound infections, it is beneficial if the material has a porous network structure because this enables the potential transfer of antibiotics or other medicines into the wound, as well as helps to prevent additional infection, and serves as a physical barrier for other microorganisms [47]. NC has an ideal structure but has no antimicrobial activity itself. Therefore, it has been combined with other biologically active molecules, including other polysaccharides, proteins, glycosides, cytokines, growth factors, local anesthetics, and even nanoparticles [26,33,64].

Depending on the used antimicrobial agent for conjunction, the nanocellulose-based antimicrobial materials are, thus, divided into two groups [65], depending on the type of incorporated antimicrobial agents, i.e., inorganic [23,66,67,68,69,70] and organic [71,72,73,74,75,76,77] materials.

This article is, thus, a review of studies published over the last five years on nanocellulose-based drug and antimicrobial agent delivery systems, by covering both “standard” inorganic drugs and antimicrobial agents (e.g., silver nanoparticles), as well as alternatives to them that are obtained from natural sources and exhibit better biocompatibility and lower toxicity. Such nanocellulose based natural hybrids also have a high potential to reduce microbial growth and, thus, could be further used in food, medical and pharmaceutical applications, as well as may help to prevent the spread of various infectious diseases.

## 2. Application of Nanocellulose in Drug Delivery

A drug can be delivered by swallowing, by inhalation, by absorption, through the skin, or by intravenous injection to produce a systemic pharmacological effect. The oral route is the most common route of drug delivery to patients, where a drug is swallowed. The problem, however, is that it is covered by low bioavailability and poor absorption of protein and peptide drugs, for what is responsible for enzymatic degradation and high molecular composition of these drugs, which results in low membrane permeability [78]. Drugs are usually retained for a short period on the mucous membranes, and the rate of diffusion through the mucous surfaces is limited, so the bioavailability of the drugs is lower. One of the key factors is the amount of time the active substance must be absorbed. Mucoadhesive drug delivery systems are often used to improve the therapeutic efficacy of the drugs. For an active substance to be absorbed in the body, it must pass through mucous membranes. Well-known routes of mucoadhesive drug delivery systems are buccal, nasal, ocular, gastro, vaginal, and rectal [79,80,81]. Further, inhalation therapy is one of the best options for pulmonary delivery as a route for systemic administration. Aerosols are used for the delivery of therapeutic agents [82]. Intravenous infusion is also commonly used for the delivery of analgesics, sedatives, antibiotics, chemotherapy agents, hormones, anesthetics, and other fluids to patients [83]. Delivery of drugs via the skin, the transdermal route, has many advantages, such as a relatively large area for absorption, less frequent dosing, noninvasive nature and avoidance of first-pass metabolism [84]. The outer layer of the skin acts as a physical barrier, so only particular molecules with suitable physicochemical properties can penetrate the skin [85]. The solution to increasing the rate of drug delivery across the skin is chemical penetration enhancers, which are also often involved in improving the bioavailability of drugs [86,87].

It is well known that cellulose has been used successfully in the US Food and Drug Administration (FDA) approved drugs or products [88]. Due to the essential properties of nanocellulose, such as high crystallinity, biocompatibility, biodegradability, high surface area, unique mechanical and rheological properties, morphology and geometrical dimensions [2,17,89,90,91], it has become widely researched for drug delivery systems in recent years. The potential multifunctionality as regards chemical modifications could be used to bind and release various therapeutic agents [92,93]. Over the last decade, nanocellulose has become extremely attractive and used in many drug delivery studies. CNFs, CNCs, and BC are quite similar, but based on certain differences and characteristics, each type of NC is better suited for a particular drug delivery system [94]. Moreover, the release time of NC-based systems varies from a few minutes to several days or months [6,95,96].

In 2014, Jorfi and Foster [55] published a review of advances in NC for biomedical applications and have covered many important applications of NC in drug delivery systems by then. Further, Plackett et al. [97] published a review of NC as a vehicle for drug delivery by covering CNC, CNF, and BC in drug delivery systems published until 2014. Later in 2019, Kiliona et al. [98] collected various modification agents for CNC and CNF-based delivery systems with different model drugs, entrapping interactions and potential applications. In addition, Salimi et al. [96] gathered literature about NC applications in oral, transdermal and local drug delivery. Because things are moving very fast in this research area, Section 2.1, Section 2.2, and Section 2.3 summarize the applications (Table 1, Table 2 and Table 3) of NC in drug delivery systems over the last five years.

### 2.1. CNCs as a Vehicle for Drug Delivery

**Figure 4:**
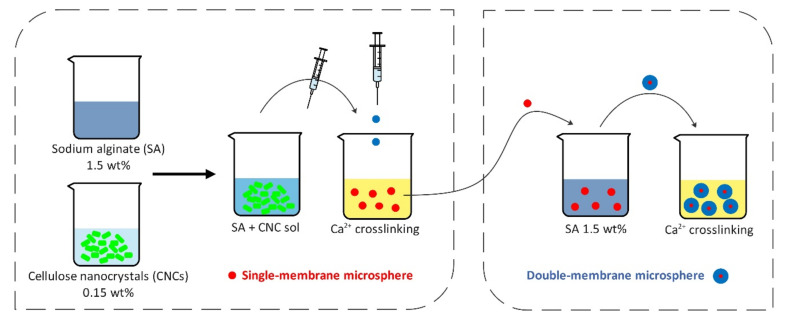
Preparation procedure of single-membrane and double-membrane microsphere hydrogels [99].

Lin et al. [99] developed biocompatible double-membrane hydrogels made from CNCs and alginate, according to the preparation method depicted in Figure 4.

Such hydrogels can release one drug quickly and another drug slowly, or, in other words, co-delivery of drugs is possible. For potential new anticancer drug delivery systems, Ntoutoume et al. [100] developed complexes from CNCs and cyclodextrin and loaded them with curcumin. Developed complexes have an antiproliferative effect on colon and rectal cancer cell lines. You et al. [101] cross-linked nanocomposite hydrogels based on quaternized cellulose (QC) and cationic CNCs with β-glycerophosphate (β-GP). Doxorubicin has been encapsulated into QC/CNC/β-GP hydrogels, and such hydrogels exhibited potential for applications in the subcutaneous delivery of anticancer drugs. Golshan et al. [102] prepared poly(propylene imine) dendrimer-grafted CNC nanostructures and conjugated these NPs with folic acid (FA) to investigate its bioconjugation effect on doxorubicin release behavior. FA conjugation prevented doxorubicin molecules from leaving the nanoconjugate system. Gorgieva et al. [103] conjugated sodium alendronate (Aln) and 3-aminopropylphosphoric acid (ApA) covalently to CNCs. These molecules contain bisphosphonates groups, which are used in drugs for the treatment of some bone diseases. With confocal microscopy, fluorescent labeling was performed with Rhodamine B Iso Thiocyanate (RBITC). Aln/Apa-modified CNCs did show much potential in drug delivery for bone cell-related diseases. Supramaniam et al. [104] synthesized magnetic CNCs (m-CNCs) and merged them with alginate. The hydrogel was loaded with ibuprofen as a model drug. The physical and mechanical properties of alginate hydrogel beads improved due to the presence of m-CNCs, which was reflected in the increased swelling degree and decreased ibuprofen release rate. In another study [105], poly(vinyl alcohol) (PVA) was reinforced with CNCs to obtain self-standing hydrogels. To prove the possibility of using hydrogels in applications for ophthalmic use, hydrogel lenses were loaded with chitosan-poly(acrylic acid) NPs and exposed to lysozyme, which is present in the eye. Md Abu et al. [106] studied the use of NC as an antimicrobial drug delivery system for honey and its application for wound healing. Due to released kinetics and good antimicrobial activity, honey incorporated CNC films could be used as wound dressings.

Many studies did investigate the binding of curcumin on NC materials. For example, Zainuddin et al. [107] extracted CNCs from the kenaf bast fibers and modified them with the cationic surfactant cetyltrimethylammonium bromide (CTAB). Further, curcumin was bound so that the curcumin/CTAB-CNC suspension was centrifuged, and a pellet was formed. It was found that the amount of curcumin bound was decreased with increasing the concentration of CTAB. Tong and colleagues [108] studied the use of CNC films for antimicrobial drug delivery in a diabetic wound dressing. Curcumin was added for the long-lasting antimicrobial effect of the film. Further, Gunathulake, Ching and colleagues [109,110,111] loaded curcumin into chitosan/CNC hydrogel. The nonionic surfactant medium (Tween 20) increased the drug loading capacity of NC compared to the drug loading capacity in the methanolic medium. For preserving drug activity, it is crucial that curcumin retains its structural integrity after release to simulated gastric fluid (SGF) and phosphate-buffered saline (PBS).

Furthermore, Li et al. [112] prepared CNCs with folate, cis-acetonitrile-doxorubicin, and polyethyleneimine. Hybrids released 95% of doxorubicin in 24 h at pH 5.5. Hivechi et al. [113] synthesized CNC-incorporated poly(ε-caprolactone) (PCL) nanofibers and studied its drug release behavior. Tetracycline was used as a model drug, resulting in controlled drug release. Drug release was slowed with an increasing amount of CNC in the PCL nanofibers.

### 2.2. CNFs as a Vehicle for Drug Delivery

Löbmann and Svagan [114,115,116] reported about CNFs used in drug formulations, with a focus on poorly soluble drugs. CNFs are used as stabilizers for achieving long-lasting sustained release and serves as film and foam formers.

Bhandari et al. [117] studied CNF aerogels as carriers for oral controlled drug delivery systems. Aerogels exhibited favorable floatability and mucoadhesive properties and were prepared with the lyophilization method. Paukkonen et al. [118] developed emulsion for immediate and sustained drug release applications. The emulsion was made from natural biopolymers, Class II hydrophobin protein HFBII from *Trichoderma reesei* and NFCs. Further, Svagan et al. [119] prepared floating solid CNF-based nanofoams loaded with furosemide. Foams exhibited sustained release of a model drug, which, in turn, depended on the drug loading, foam dimension, as well as the solid-state of the drug. Fakhri et al. [120] developed Fe_3_O_4_-Ag_2_O quantum dots/CNF nanocomposites and grafted them with two anticancer drugs. The results of the study showed that nanocomposites could be applied to the drug delivery system for treating skin cancer. Paukkonen et al. [121] used anionic CNF hydrogels for the delivery of small molecules and proteins. Freeze-drying into aerogels and redispersion into hydrogels of the before mentioned hydrogels is also possible. Guo and colleagues [122] fabricated CNF/alginate beads for the release of metformin hydrochloride. CNFs improved encapsulation efficiency and enabled more sustained drug release. Poonguzhali et al. [123] prepared alginate and CNF film loaded with ampicillin and investigated in vitro drug release. NC and ampicillin did improve the swelling and mechanical properties of alginate; moreover, ampicillin-loaded films exhibited good drug delivery systems with sustained drug release. Liu and colleagues [124] combined CNF and polydopamine (PDA) with calcium ion as a cross-linker. Tetracycline hydrochloride (TH) was used as a model drug for testing hydrogel as potential drug delivery carriers. In vivo skin defect experiments showed the synergistic effect of hydrogel on promoting wound healing. Sarkar, Orasugh et al. [125] prepared CNF/chitosan (CNF/CS) film with potential use in transdermal delivery. The film was loaded with an anti-inflammatory and analgesic agent, ketorolac tromethamine (KT). Drug release of KT was sustained with the incorporation of CFNs, and, therefore, this nanocarrier could be a potential candidate for transdermal drug delivery systems. Further, Orasugh et al. [126] formulated a nanocomposite of CNF and hydroxypropylmethylcellulose (HPMC). The nanocomposite was loaded with KT. Moreover, in vitro drug release results showed that the cumulative drug release rate decreased with the increase of CNF concentration in nanocomposites. An aim of Auvinen and colleagues’ [127] study was to modulate the drug release properties of CNF hydrogel. To limit the direction of drug diffusion, the first step of their study was to manufacture non-active capsules with the 3D printing method, using poly(lactic acid) (PLA). Next, drug dispersion was made of model compounds (beta-blocker metoprolol and nadolol) and anionic CNF hydrogel. The results showed that, by adjusting the geometry of the 3D printed PLA capsule, sustained release profiles provided by the CNF matrix could be modulated accurately. Importantly, however, the release of any CNF-compatible drug can be modulated easily by altering the inner geometry of the PLA capsule. Further, CNF and gelatin cryogels (hydrogels, synthesized at subzero temperatures [128]) were used for delivery of the widely used compound for treating cancer, 5-fluorouracil [129]. The structure of cryogels, CNF/gelatin ratio, density, cross-linking degree, and pH values all influence the behaviors of drug release. The duration of the sustained drug release time is up to 12 h. Meneguin et al. [130] reinforced starch/pectin free-standing films with CNFs or BNC fibers, and tested them for colonic methotrexate release. The study showed that CNFs are the best nanofiller, as the addition of CNFs improved the mucoadhesive, barrier, mechanical, and release properties of films. Such films are promising as poor solubility drugs carriers, as they increased drug dissolution rates, with approx. 80% of methotrexate release in 2 h and 30 min.

Fiorati and coworkers [131] tested the mechanical and drug release characteristics of aerogels made from cross-linked CNFs using amine-containing polymers and citric acid. Ibuprofen and amoxicillin were used as model drugs, and the results showed good absorbing properties for possible drug delivery applications. Recently, however, the same group of researchers [132] made stable hydrogels from TEMPO-CNFs with added Ca^2+^ ions. Proposed hydrogels are suitable for control drug delivery of ibuprofen and are proven to be cytocompatible. In particular, it is interesting to note that the addition of a certain amount of polyvalent cation can adapt the physicochemical properties of the material, and the formation of self-sustaining hydrogels is possible.

### 2.3. BNC as a Vehicle for Drug Delivery

BNC is a widely explored nanostructured matrix in different applications and is used in many fields, such as the food industry, drug delivery, biomedical materials, nanostructured biomaterials, and Tissue Engineering [24].

Alkhatib et al. [133] produced a BNC/poloxamer hybrid system that provides prolonged retention time for the octenidine, an antiseptic drug. An aim of the next study [134] was to develop fish scale-BNC biopolymer composite microneedles and load them with lidocaine, the medication most often used for numbing tissue in a specific location. Prepared microneedles could pierce the outermost layer of the epidermis and dissolve in the skin to release loaded drugs, in this case, lidocaine. Further, a group of researchers [135] developed pH-sensitive systems for the controlled release of diclofenac based on poly(*N*-methacryloyl glycine)/BNC composites, whose thermal, mechanical and viscoelastic properties were good. These nanocomposites have high water uptake capacity, are non-cytotoxic and pH-sensitive (the drug was released at pH 7.4, while at pH 2.1, the drug was retained in the nanocomposite) and, therefore, also suitable as carriers for dermal and oral administration. Ratnayake et al. [136] investigated freeze-dried BNC as a matrix for controlled protein delivery. Due to its high-water solubility, abundance, and good stability, bovine Serum Albumin (BSA) has been used as a model drug. The results of the study showed a good pattern for the loading and release profiles in the system used and, moreover, research indicates the importance of BNC as a carrier for protein drugs. Fey at al. [137] showed that BNC is suitable as a carrier for intestinal epithelial cells in drug delivery studies. Further, Silva et al. [138] investigated long-term storage of BNC membranes, loaded with lipophilic and hydrophilic active pharmaceutical ingredients (APIs). They evaluated stability at different temperatures and relative humidity. The results of the study showed that all BNC membranes loaded with APIs were stable and did not change either structurally or morphologically. Due to the hydrophilic nature of BNC membranes, the moisture-uptake increased with relative humidity. Meanwhile, Abba et al. [139] studied the incorporation of crocin (water-soluble pigmented carotenoid of saffron, which possesses antioxidant, antitumor, memory enhancer, antidepressant, anxiolytic and aphrodisiac properties [140]) into a BNC membrane. The results of direct dissolution and transdermal pass have shown compelling release, making BNC membranes a promising way for delivery of crocin.

## 3. Nanocellulose Based Antimicrobial Hybrids and the Use of Antimicrobials in Drug Delivery

It is important to note that native (as produced) NC does not possess antimicrobial properties. This can be achieved by functionalizing it or incorporating antimicrobial agents [55].

Halogens, phenols, silver nanoparticles and quaternary ammonium salts are used widely as antimicrobial agents [141]. Silver has been used most extensively for healing infections because its nanoparticles have proved to have antimicrobial characteristics [142]. Metal oxide nanoparticles (TiO_2_, CuO, ZnO, MgO) also exhibit antimicrobial activity [65]. For less toxic and more sustainable effect, some organic antimicrobial agents, such as porphyrin, lysozyme, lactoperoxidase, lactoferrin, chitosan-benzalkonium chloride, chitosan-methylisothiazolinone, gentamicin, ɛ-polylysine and sorbic acid were incorporated in NC [47,72,143,144,145]. These nanomaterials have promising antimicrobial properties against Gram-positive and Gram-negative bacteria.

An unexplored and unknown area is the balance between better antimicrobial activity, the duration of its effect and control of human cell damage [6].

### 3.1. Inorganic Hybrids

New therapeutic agents are also based on the use of metal nanoparticles that have an antimicrobial effect. Most commonly used and researched are silver nanoparticles (AgNPs) because of their properties such as size, shape, broad spectrum of antimicrobial activity and others [146]. Mostly they are conjugated with BNC or CNFs. They are attractive for research because AgNPs did exhibit strong cytotoxicity against fungi and viruses [147], besides quality growth inhibition of bacteria such as *Staphylococcus aureus*, *Enterococcus faecalis, Escherichia coli*, *Bacillus subtilis, Klebsiella pneumonia, Vibrio cholerae, Pseudomonas aeruginosa*, and *Salmonella typhi* [146,148,149,150,151,152,153]. Furthermore, AgNPs can enter inside the bacteria, accumulate on bacteria cells’ enzymes and proteins and interact with deoxyribonucleic acid, which causes further damage and, consequently, bacteria inactivate as well [149].

**Figure 5:**
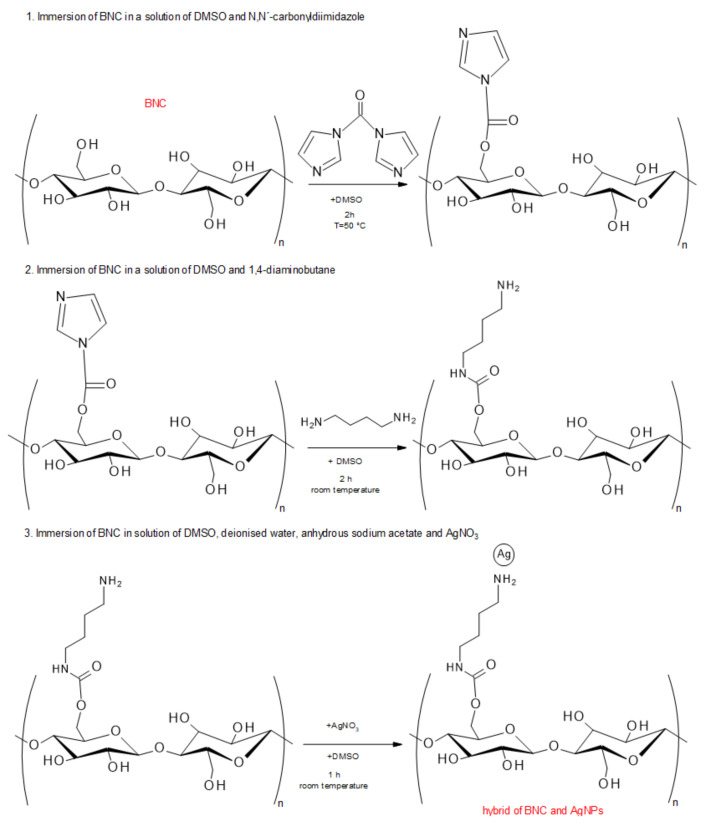
Schematic procedure to prepare hybrids consisting of BNC and AgNPs (summarized by [23]).

Various methods for the preparation of AgNPs/NC hybrids have been described [23,66,154]. Mostly used is the AgNO_3_ solution combined with strong reducing agents. NC membrane is immersed into a silver precursor (AgNO_3_), so agglomerates are immobilized only by physical interactions [155]. The schematic procedure of the preparation of BNC-AgNPs hybrids is shown in Figure 5.

The described hybrids have proved good antimicrobial activity against *B. subtilis, E. coli*, and *S. aureus* [23,65,156]. Furthermore, loading BNC membranes with Ag nanoparticles showed 99% growth inhibition of *E. coli, S. aureus* and *P. aeruginosa* [157]. The antimicrobial properties have been proven, so the connection between silver and drug delivery is interesting. Incorporation of silver ions within biocompatible and osteoconductive biomaterial hydroxyapatite (HAp) has been performed in many studies, with the option of long term silver ion release rates [158,159,160]. Furthermore, AgNPs were previously incorporated in chitosan hydrogels [161] and hybridized structures [162], thereby achieving prolonged and controlled release. The drug release rate and hydrophilic ratio also increased-with-increased Ag concentration.

As the biocompatibility of AgNPs has not yet been determined, and it is well known that AgNPs’ toxicity is concentration-dependent, the use of such hybrids in the field of Biomedicine is limited [163,164]. Therefore, Lizundia et al. [165] designed antimicrobial bio-based films composed of CNCs and metallic (Ag, ZnO, TiO_2_) nanoparticles. They all showed antibacterial effectiveness against *E. coli* and *S. aureus*.

Many metal oxide nanoparticles have already proven successful in drug delivery systems [166]. For example, as a type of pH-responsive drug carrier, ZnO nanostructured materials were suggested in 2010 [167,168]. TiO_2_ particles have also been used for such drug carriers [169], and later these materials were often applied for drug delivery systems [170]. The beforementioned and some other studies of inorganic NC-based antimicrobial hybrids are summarized in Table 4.

### 3.2. Organic Hybrids

Natural polymers such as polysaccharides and proteins are becoming an important basis for the development of antimicrobial materials for various applications. While some biopolymers (e.g., chitosan and lysozyme) are natural biocides, others (e.g., cellulose) must be bind to bioactive compounds to obtain such properties [179].

Among many applications (e.g., reinforcing agents in nanocomposites, biodegradable films, barriers for packaging, stabilizing agents in dispersions for technical films and membranes, additives in food, texturing agents in cosmetics [180]), CNFs have been used to prepare thin films for wound dressing and bioactive implants [181]. Most NC applications in the Biomedical field are still progressing in the laboratory, assessing its ability, capacity, reproducibility, and effectiveness in providing its roles as scaffolding and regeneration, pharmaceutical applications (e.g., drug carrier) and implants [182,183,184,185].

The biggest problem with the wound healing process is a secondary infection, where bacterial sepsis can be fatal in the case of some extreme burns. Because NC itself has no antimicrobial properties, it needs to be modified [186], e.g., loading the BNC membrane with an antimicrobial agent such as benzalkonium chloride (BZC) [187] or polyhexanide (PHMB) and povidone-iodine (PI) [188]. BZC is an antimicrobial cationic surfactant, which was used widely in commercial wound dressings. It effectively inhibited the growth of various bacteria (*E. coli, S. typhimurium, B. subtilis*). Previously, BZC has already been used in drug delivery studies [189,190,191]. For example, Garcia-Fernandez et al. [192] prepared cyclodextrin-functionalized hydrogels and gauzes with BZC that could be used for the prevention and management of wound infections. PI has a high molar mass and, therefore, has a delayed-release compared to PHMB. Its important feature is good biocompatibility; however, it showed less inhibition of *S. aureus* growth than BNCs with PHMB. Finally, BNC loaded with PHMB provides a better and wider therapeutic window.

The next solution could be to fabricate composites of BNC with another material (e.g., chitosan [193]) showing antibacterial properties. Wiegand et al. [188] found PHMB-loaded BNCs to be promising wound dressings exhibiting controlled drug delivery, whereas PI showed significantly delayed-release compared to PHMB.

A recent study aimed to produce and characterize an optically transparent film with good antimicrobial properties from ginger CNF, fabricated using chemicals and ultrasonication. Antimicrobial effectiveness was tested using the agar disc diffusion method. Tested microorganisms in the study were *S. aureus, E. coli, P. aeruginosa, B. subtilis*, and *C. albicans*. Chloramphenicol and nystatin were used as positive controls. They tested raw ginger fibers and both cellulose film made from ginger with chemicals (alkalization, bleaching, acid hydrolysis) and with ultrasonication. All of the samples inhibited the growth of all microorganisms. Researchers concluded that the sonication process does not damage the fiber’s natural antimicrobial properties because there was no perceptible difference in antimicrobial effectiveness between sonicated and acid hydrolyzed nanofiber films [194].

On the other hand, some studies used different ginger-derived substances (lipids [195], phenolic compounds [196,197]) in drug delivery systems. Zhang et al. [195] describe a ginger-derived nanovector made of ginger-derived lipids that can serve as a delivery platform for the therapeutic agent to treat colon cancer. In a subsequent study, Naghs [197] described the potential anti-inflammatory and anticancer action of ginger extracts jointed with nanotubes as a technique for drug delivery.

**Figure 6:**
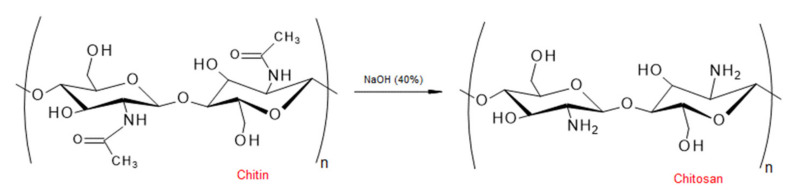
Schematic procedure of preparation of chitosan from chitin (summarized by [193,199]).

Chitosan (CS), which is derived from chitin (Figure 6) and is one of the naturally-occurring antimicrobial agents, has gained much popularity in commercial applications [198].

Due to its molecular weight, degree of deacetylation, positive charge and mucoadhesive nature [200,201,202], it is considered an efficient matrix molecule or carrier in drug delivery systems [203,204,205,206,207,208]. Precisely because of the increase in bioavailability, mucoadhesive substances (e.g., chitosan, cellulose derivatives [209]) are usually used in pharmaceutical formulations, which have an increased affinity for mucosal membranes and adhere to mucous membranes. With carefully planned mucoadhesion, specific retention of the active substance at a particular site is possible, and a controlled rate of drug release can be achieved [81].

Chitosan and its derivatives have been used widely in many biomedical applications [210], including in antimicrobial wound dressing [211], drug delivery systems [212], and tissue engineering [213].

Already in 1998, Felt and her colleagues [214] wrote of chitosan as a unique polysaccharide for drug delivery. Their article describes many applications of chitosan in the oral, injectable, nasal, ophthalmic, and transdermal routes of administration. More recent studies of drug delivery applications of chitosan and chitosan-based NPs are described in reviews from Pahri [215] and Naskar et al. [216].

**Figure 7:**
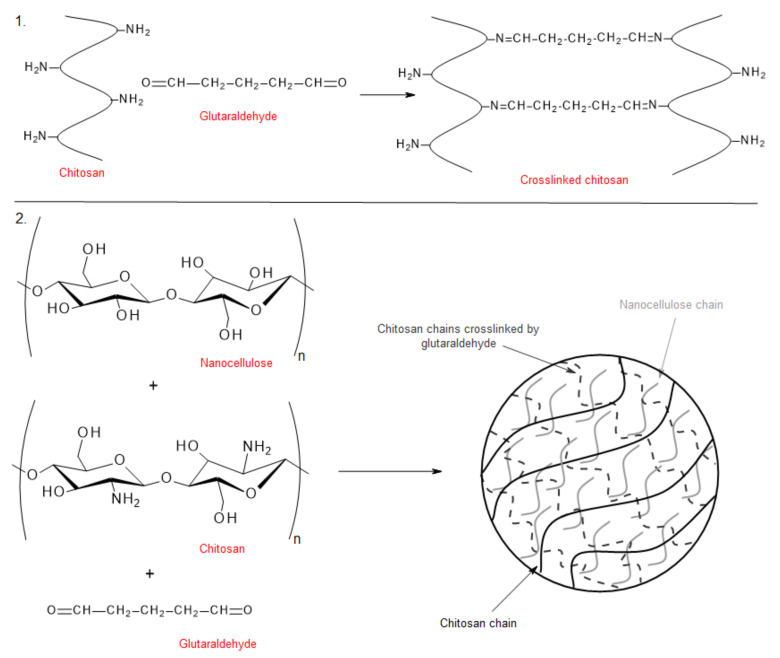
Schematic procedure for the formation of nanocellulose-chitosan hydrogel (summarized by [221]).

By adding chitosan to NC (Figure 7), the researchers improved the mechanical properties of the NC and contributed to the antimicrobial effectiveness [217] of the cellulose-based material. Chitosan is a naturally occurring amino polysaccharide with outstanding properties such as biodegradability, biocompatibility, nontoxicity, healing enhancement, and, especially, antibacterial properties [218,219].

Researchers studied the antibacterial effectiveness of hydrogel CS/BNC and lyophilized CS/BNC against *E. coli* and *S. aureus*. For the lyophilized samples, the number of bacterial colonies was significantly lower than the BNC control group. For the hydrogel samples, the bacterial numbers on all CS/BNC composites were lower than the BNC group but higher than the number just after inoculation, also showing a bacteriostatic ability. The antibacterial effect of lyophilized samples was much stronger than that of the hydrogel samples. These properties provide fabric-reinforced CS/BNC composites with great potential as excellent medical materials for wound dressings [220].

For comparison, the following study covers the use of CNCs in the synthesis of antimicrobial hybrids with chitosan. Poonguzhali et al. [222] studied chitosan polyvinylpyrrolidone (PVP)-NC (CPN) bionanocomposites for wound dressing application. PVP, polyvinyl pyrrolidone, is a non-toxic, biocompatible polymer usually applied in controlled drug release and wound dressing. It exhibits hydrophilic properties, universal solubility, and a good tendency for the formation of complexes with a wide range of molecules. Due to the latter properties, these polymeric films are attractive drug delivery systems, especially for systemic effect through the sublingual and buccal routes [223,224,225]. CPN was tested against *S. aureus*, and *P. aeruginosa* strains with modified agar diffusion assay. The largest inhibition zones were observed for the Gram-negative bacteria *P. aeruginosa*, while smaller was observed for the Gram-positive *S. aureus*. They suggest that the reason for better inhibition of Gram-negative bacteria may be that chitosan is charged positively and microbial cell membranes are charged negatively. Therefore, the interaction between them leads to bacterial leakage by disrupting the bacterial strain’s metabolism. In addition, the extremely large NC surface eased the adsorption of the target bacteria, which accelerated antimicrobial efficiency.

Furthermore, Hasan et al. [226] prepared composites from chitosan, PVP and CNCs for delivery and release of curcumin in wound dressing application. CNCs improved the thermal, swelling, and mechanical properties of the film. In addition, better protein absorption was observed in the presence of CNCs in the films, which improved the wound healing properties of the dressing material. The proposed release system is a potential dressing material in wound healing applications.

**Figure 8:**
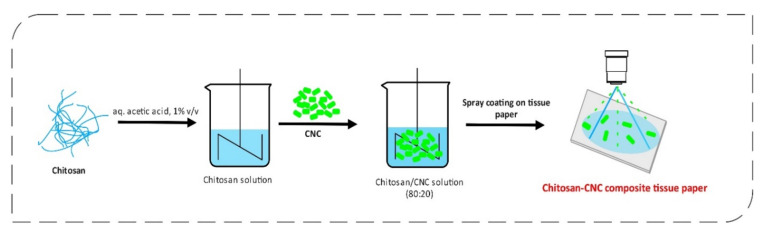
Preparation of chitosan/CNC composite tissue paper that can help prevent the spread of infectious diseases.

Recently, antimicrobial tissue paper’s potential for preventing the spread of infectious diseases has been developed. Potential inhibition of the spread of COVID-19 has also been reported [227,228], but these claims are not substantiated. To confirm these speculations, antimicrobial tissue paper should be tested specifically against COVID-19 to determine the effectiveness of these materials. Researchers prepared hand-sheets which have been spray-coated with chitosan, CNC, and their composite coating (chitosan-CNC). The main purpose of the study from Tyagi et al. [229] was to empower tissue paper composites with a synergistic effect of CNC and chitosan on increased water absorbency, mechanical strength, and antimicrobial activity. Tissue paper sheets were made with the use of a handsheet mold from recycled, bleached, deinked pulp. Chitosan solution was prepared in 1% acetic acid, which was further mixed with CNC for 1 h at 500 rpm. The ratio of chitosan/CNC was 80:20 by weight. Before surface coating, researchers conditioned the handsheets at 50% relative humidity and 23 °C for 24 h. chitosan-CNC coatings (1% solid concentration) were spray-coated on the handsheets and further dried at 110 °C. The preparation of such antimicrobial tissue papers is depicted in Figure 8.

Furthermore, tissues exhibited antimicrobial activity against *E. coli*, the growth of which was inhibited by up to 98%. chitosan-CNC coatings exhibited a zone of inhibition with a diameter of 17.4 mm. The synergistic effect of chitosan and CNC proved to be excellent, as coatings with chitosan alone showed an 8.7 mm zone of inhibition. The coatings were also improved by plasma treatment, as the plasma-treated chitosan-CNC coated tissue paper exhibited the highest zone of inhibition, with a diameter of 23.4 mm and a 99% reduction in the growth of *E. coli.* Plasma-treated coatings additionally showed antimicrobial activity against microbes from a human–hand sample, in which the presence of many different bacteria, fungi and virus is expected. The atmospheric plasma used was composed of helium as an inert gas and oxygen as a reactive gas, and certainly, oxygen plasma is known to increase the hydrophilic nature of tissue paper, resulting in increased antimicrobial activity of plasma-treated chitosan-CNC coated tissue paper [227,228,229,230].

Not only BNC and CNCs, but CNFs are also used in combination with chitosan and other active ingredients to achieve an antimicrobial effect. For packaging applications, Sundaram et al. [231] developed biodegradable composite membranes with antimicrobial properties. They combined CNFs, chitosan and S-nitroso-N-acetyl-D-penicillamine (SNAP). The zone of inhibition showed against *S. aureus*, *E. faecalis*, and *L. monocytogenes*. The point is that the SNAP molecule contains a group of nitric oxide (NO), which inhibits the growth of bacteria (both Gram-positive and Gram-negative), viruses, fungi, and yeast [232]. In addition, it is an endogenous antiplatelet agent, and therefore the exogenous release of NO from different polymer matrices has shown the reduction of thrombosis and infection of implantable medical devices [233].

A recent study examined the antimicrobial effectiveness of NC and grape seed extract added chitosan and polycaprolactone (PCL) based biofilms [234]. Grape seed extract contains many phenolics, such as catechins, epicatechin, gallic acid, and procyanidins. These have shown various biological effects, including antimicrobial abilities [235]. Further, PCL is an aliphatic polyester, and when mixed with chitosan, allows it to maintain its mechanical properties and improve its barrier properties. Films like that could be used in food packaging applications. All film samples showed antimicrobial activity against *E. coli* and *Listeria monocytogenes*. The high permeability of PCL caused by its rubbery characteristics enables the delivery of low molecular weight drugs such as vaccines [236] and steroids [237]. Compared to other biodegradable polyesters, PCL has become interesting in drug delivery due to its lack of toxicity and low cost. However, due to its high crystallinity and hydrophobicity, its deficiency is a slow degradation rate in vivo.

The solution, however, is that PCL biodegradability can be increased by copolymerization or by blending it with other polymers. Therefore, Sahoo et al. [238] blended chitosan with PCL for controlled delivery of ofloxacin. In addition to all the other good properties of chitosan (e.g., low cost, availability, positive charge, biocompatibility, and antimicrobial activity), this blend is a more biodegradable and biocompatible material that can be used in controlled drug delivery systems.

The production of antimicrobial NC membranes can be achieved by chemical grafting of functional groups on the surface of cellulose nanofibers (e.g., CNF functionalized with amino and aminosilane groups, which inhibited the growth of *E. coli* and *S. aureus*) [141,239,240].

For example, a chemical grafting of aminoalkyl groups onto the surface of BNC membranes may be used to mimic the internal antimicrobial properties of chitosan. The presence of free amino groups along the polymer chain is responsible for the antimicrobial efficacy of chitosan.

**Figure 9:**
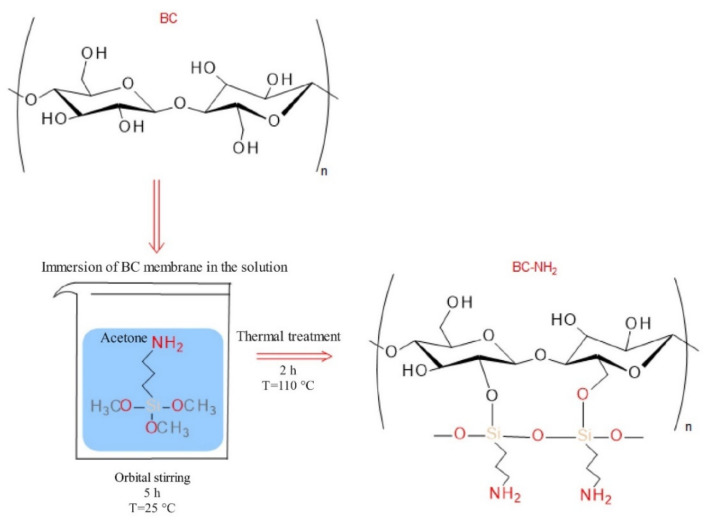
General procedure for bacterial cellulose silane chemical grafting with APS (summarized by [73]).

Fernandes and Sadocco et al. [73] used 3-aminopropyltrimethoxysilan (ASP) to achieve chemically grafted aminoalkyl groups at the surface of BNC (Figure 9). They tested antimicrobial activity against *S. aureus* and *E. coli* strains using a standard dynamic shake flask method. BNC-NH_2_ membranes showed a significant reduction in bacterial viability for both *E. coli* and *S. aureus*. A study showed that the silane chemical grafting approach produces a BNC membrane with antimicrobial activity while maintaining biocompatibility.

Furthermore, BNC is a highly pure form of cellulose and a swollen membrane with high water content [241]. However, due to its physical and mechanical properties, this biopolymer is of great interest for use in the Biomedical field, and BNC-NH_2_ membranes have potential for applications in tissue engineering, wound healing, and drug delivery systems.

The purpose of the following study was to evaluate the antimicrobial activity of BNCs filled with nisin. Nisin is a 34-amino acid long bacteriocin, which is active against many foodborne bacteria [242]. BNC membranes were immersed into a nisin solution with or without EDTA, kept in a shaker and then immersed in phosphate buffer solution (PBS). The antimicrobial activity of nisin, whether using EDTA solution or not, was assessed by minimal inhibitory concentration (MIC) and the agar diffusion method. Tested microorganisms were *S. aureus, E. coli*, and *P. aeruginosa*. A combination of nisin solution with EDTA showed a synergistic effect. For *E. coli* and *S. aureus*, antimicrobial activity was observed, while for *P. aeruginosa*, it was not. BNC membranes loaded with nisin are promising agents to prevent microorganism contamination [243].

The delivery of nisin for antimicrobial efficacy has been investigated extensively in recent years. Ugurlu et al. [244] found out that an envelope from pectin and hydroxypropyl methylcellulose (HPMC) is a good delivery system for nisin to be delivered to the colon. Further, Coelho Correia et al. [245] found a poly(lactic-co-glycolic acid) (PLGA)-nisin matrix promising for sustained drug delivery, with continuous release of nisin for two weeks. Nisin embedded in polymer matrices has, thus, the potential for topical drug delivery. Shin and colleagues [246] described various biomedical applications and delivery of nisin, including the treatment of various infections, mastitis, oral health, and cancer.

Further, BNC was loaded with bromelain (BL). That is a protease found in pineapple tissues [247,248]. bromelain was incorporated in BNC membranes by immerging membranes in a bromelain solution. The bromelain antimicrobial activity is related to its enzymatic activity. They tested the initial bromelain solution, residual bromelain solution, and bromelain solution after being released from BNCs. Results showed that it inhibited all tested microorganisms (*E. coli, S. aureus*, and *P. aeruginosa*), especially for the release solution.

In addition, the BNC-BL membrane proved to be an auspicious drug delivery system that shortens healing time, reduces the risk of infections, and helps relieve pain [249]. Bagga et al. [250] used bromelain capped gold NPs as a drug delivery carrier of the antibiotic levofloxacin. Due mainly to the stability of such NPs and the delivery of a large number of levofloxacin molecules to a highly localized area at the point of contact between the NPs and the bacteria, the NPs have proven as effective carriers of the selected antibiotic and, further, improved antimicrobial activity against Gram-positive and Gram-negative bacteria. BL is known as a phytotherapeutic anticancer agent [251], but its activity is decreased upon oral administration, and for this reason, Bhatnagar et al. [252] encapsulated BL into PLGA to formulate NPs and coated them with polymer for stability against acidic gastric conditions. Based on the generation of reactive oxygen species, induction of apoptosis and weakened mitochondrial membrane potential in Ehrlich cells (in mice), these NPs are potential candidates for oral chemotherapy.

Enzybiotics are particularly interesting. They have been proclaimed as an environmentally safe and interesting alternative to antibiotics as antimicrobial agents. Today, all enzymes with antibacterial and antifungal properties are included in the enzybiotic group [253].

Antimicrobial activity of crude laccase against Gram-positive and Gram-negative bacteria has been observed, which is interesting because laccase is known to catalyze reactions, leading to the emergence of antimicrobial species. BNC membranes were lyophilized and immersed in the laccase preparation diluted in a phosphate-citrate buffer. The antimicrobial effect was evaluated against *S. aureus* and *E. coli*. Antimicrobial activity was compared with Ag nanoparticles (AgNPs), and Laccase/AgNPs immobilized on BNC. The results showed the antimicrobial effect of the laccase. Moreover, laccase inhibited the growth of Gram-positive bacteria better than Gram-negative ones (about 92% (*S. aureus*) and 26% (*E. coli*) of bacterial inhibition) [254].

Lacasse was not observed in literature for drug delivery systems, but recently, Zhang and colleagues [255] revealed a food gel of soluble crosslinked corn bran arabinoxylan (CAX). Alkali-extracted CAX was treated with laccase to form soluble crosslinked CAX, which formed a gel on pH reduction. Such gels could be taken in the low pH environment of the stomach (e.g., as food gels or beverages containing soluble crosslinked CAX) and as a drug delivery matrix.

After a few listed applications with BNC, the following study [108] used CNC film as a wound dressing material and conjugated it with curcumin. It is a lipophilic phytopolyphenol isolated from *Curcuma longa*. With the controlled release of curcumin, they wanted to achieve a long-lasting antimicrobial effect of the NC film. They used the agar diffusion test and Hohenstein challenge test (AATCC-100) as indicators for antimicrobial activity. The results of the study were great, as curcumin loaded film showed significant antimicrobial activity on three Gram-positive (methicillin-resistant *S. aureus*, *Streptococcus* sp., *Bacillus coagulans*), two Gram-negative (*E. coli*, *Proteus mirabilis*) bacteria and one yeast (*C. albicans*). Curcumin-loaded film reduced the growth of all tested microbial cells significantly, as five of six test microorganisms showed a 99% growth reduction relative to the control.

Furthermore, Raghavendra et al. [256] impregnated nanocurcumin into gelatin nanocellulose fibers for potential antimicrobial activity and further applications. Study results showed antimicrobial activity against *E. coli* and *S. aureus* for 24 h. Moreover, curcumin possesses chemopreventive, chemotherapeutic, antioxidant, anti-inflammatory and hyperlipidemic properties [257].

Due to poor aqueous solubility and poor oral bioavailability, the therapeutic efficacy of curcumin is limited [258], but it is still used widely in applications for drug delivery systems because of its outstanding antimicrobial properties. For example, Sanoj Rejinold et al. [259] formulated highly stable thermoresponsive nano constructs of curcumin with chitosan-g-poly (N-isopropylacrylamide) for drug delivery, Sun et al. [260] used exosomes, released from a number of different cell types, as a vehicle for curcumin delivery, and Pham et al. [261] modified curcumin-loaded superparamagnetic iron oxide NPs with chitosan for use as drug delivery carriers in the treatment of cancer cells. Recently, Gupta et al. [262] published a review of the latest curcumin-based nanoformulations for targeting different diseases.

Improving the properties of CNCs and adding antimicrobial activity to them can be done by esterification of the surface of CNCs using non-toxic resin acids, rosin (colophony; a natural product of coniferous trees [263]). This was proven by de Castro et al. [264]. The rosin-grafted CNC showed strong antibacterial effectiveness against Gram-negative bacteria (*E. coli*) and small antibacterial activity against Gram-positive bacteria (*B. subtilis*).

Rosin is one of the highly stable, non-toxic, biodegradable, and gel-forming natural gums. It possesses film-forming and coating ability, and further, it has been used as microencapsulating agents and as anhydrous binders, or matrix material in tablets for controlled drug release [265]. Kumar Yadav et al. [266] described and reviewed pharmaceutical applications of rosin, including film-forming materials, transdermal drug delivery, and targeted drug delivery. It was used mainly as a taste-making agent, microencapsulating agent and material, binding agent, matrix-forming material, and emulsifying agent for achieving good stability, faster dissolving and sustained controlled, or sustained drug release.

Preparation and investigation of antibacterial materials by utilizing protein degrading enzymes (e.g., lysozyme) is also interesting. Such enzymes degrade bacteria by adherence [267].

Lysozyme is antimicrobially effective against Gram-positive and Gram-negative bacteria [268]. Uddin, Orlema et al. [267] investigated the antibacterial activity of CNF-lysozyme aerogels and compared them with silver-containing CNF aerogels. Lysozyme aerogels showed antibacterial effect at *E. coli* and *S. aureus* strains, as did silver CNF aerogels, which antibacterial activity depended on the nanoparticle concentration. It was observed that the higher the Ag nanoparticle concentration, the higher the inhibition of the microbial culture.

Besides antimicrobial activity, lysozyme exhibits antitumor, antiviral and immune-modulatory activities; therefore, having the ability to suppress tumor cells [269]. Alves et al. [270] studied the potential use of fluorinated ionic liquids (FILs) as a drug delivery system for proteins. They used lysozyme as a model protein and investigated the impact of FILs on its structure and function. Its hydrolytic performance, therefore, its stability and activity did not decrease. Further, Wu et al. [271] prepared chitosan/sodium alginate-based (CS-ALG) hydrogels for the delivery of lysozyme. Antibacterial activity of hydrogels was improved with the addition of lysozyme, and the relative activity of the released lysozyme was 87.72 % ± 3.96%, which makes CS-ALG hydrogels a promising matrix for enzyme loading and their delivery.

**Figure 10:**
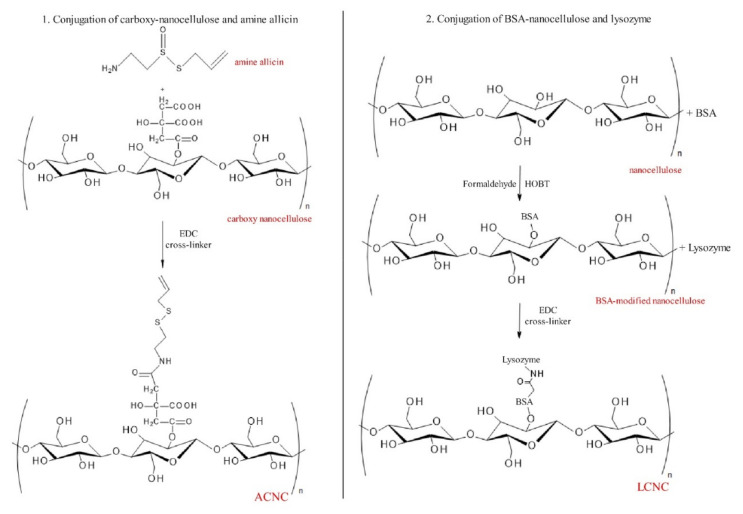
Schematic procedure of conjugation between nanocellulose, allicin, and lysozyme (summarized by [274]).

Allicin is an antimicrobial agent that forms when a clove of garlic is freshly crushed. It is formed when the enzyme alliinase gets in contact with alliin and converts it to allicin [272]. Jamindar et al. [273] formulated and characterized allicin-amphotericin-B liposomal gel for topical treatment of fungal infections, but a recent study from Jebali and coworkers [274] describes the antimicrobial activity of cellulose NPs conjugated with allicin and lysozyme separately (Figure 10).

Antimicrobial properties were determined with the microdilution method and compared to allicin, lysozyme, and CNC alone. The allicin and lysozyme were conjugated to CNC by a carbodiimide cross-linker, both showing good antifungal and antibacterial effects against *Aspergillus niger*, *C. albicans*, *S. aureus*, and *E. coli* [274,275].

Atef et al. [276] incorporated summer savory (*Satureja hortensis L.* [277]) essential oil into agar-based nanocellulose films. They evaluated antimicrobial activity with the disc diffusion method against *L. monocytogenes, S. aureus, B. cereus*, and *E. coli*. Samples without savory essential oil (SEO) were considered as control and did not exhibit any zone of inhibition. The addition of SEO showed a weak inhibitory effect. *L. monocytogenes* and *B. cereus* were the most sensitive microorganisms to the SEO containing nanofilms, and *E. coli* was the most resistant bacterium. As for delivery systems, SEO was not used, except when Cansu Feyzioglu and Tornuk [278] formulated chitosan NPs loaded with SEO for potential antioxidant and antimicrobial delivery applications.

The next few antimicrobials were conjugated with cellulose and also have great potential for conjugation with NC materials. As a result, they could be good NC-based antimicrobial hybrids, acting as an alternative to classic antimicrobials.

One of the natural antibiotics is propolis. Conjugated with BC, it shows potent antimicrobial activity and wound healing properties [279,280]. Ethanol extracts of propolis can inhibit the growth of *S. aureus*, *Enterococcus* sp. and *P. aeruginosa* [281].

Propolis has been involved widely in research on drug delivery systems. In 2010 Dantas et al. [282] developed propolis microemulsion for topical applications. Ahmed et al. [283] found out that Egyptian propolis extract in a cold cream formulation, alone or in combination with Dermazine, is appropriate for topical administration for the treatment of diabetic burn wounds. Further, Berretta et al. [284] created a formulation with 3.6% propolis extract, which can benefit in the treatment of skin injuries. Balata et al. [285] formulated pluronic lecithin organogel with propolis that exhibited great skin permeation and antimicrobial activity, and later, Rassu et al. [286] prepared propolis as lipid bioactive nanocarrier. Propolis-based solid lipid NPs could be used as delivery systems of drug and flavonoids and for the local treatment of nasal diseases.

Honey from the Manuka tree conjugated with BC also showed antibacterial activity. Its quality is to promote tissue growth, so Manuka honey is one of the promising agents in wound healing applications [287]. Drug delivery dressings are defined as substances applied directly to the surface of the wound to display therapeutic properties in terms of benefit to the healing process. Manuka honey could be a therapeutic agent and vehicle itself and can be compared to many other natural therapeutic polymers (e.g., alginate, chitosan, collagen, and hyaluronic acid) used as drug delivery dressings [288,289]. Tenci et al. [290] developed a powder formulation for delivery of manuka honey bioactive components and platelet lysate (PL) in chronic skin ulcers.

Cinnamaldehyde and eugenol (the main ingredients of cinnamon [291] and cloves [292]) were incorporated with cellulose films to obtain active antimicrobial materials. Antimicrobial effectiveness was investigated against nine bacteria (*B. cereus, E. faecalis, L. monocytogenes, Micrococcus luteus, S. aureus, Aeromonas hydrophila, E. coli, P. aeruginosa, Salmonella enteritidis*) and three strains of yeast (*C. albicans, Saccharomyces cerevisiae, Zygosaccharomyces rouxii*). The agar well diffusion method and a vapor diffusion technique were used for its determination. *A. hydrophila* and *E. faecalis* were the most sensitive to cinnamaldehyde or eugenol films. The cellulose-based film containing cinnamaldehyde or eugenol did not completely inhibit the growth of microbial cells. However, they showed positive activity against all selected strains in terms of size and number of microbial colonies in the vapor diffusion test. This study demonstrates the potential use of cinnamaldehyde and eugenol for application to an antimicrobial film or coating [293].

Recently, Kenawy et al. [294] combined gelatin, chitosan, and cinnamaldehyde and developed biodegradable crosslinked membranes that demonstrate the potential as antimicrobial dressings for mitigating wound healing, and, interestingly, Bang and Kim [295] developed a stable self-microemulsifying drug delivery system for trans-cinnamaldehyde use in the Pharmaceutical industry.

Also, eugenol is used widely in drug delivery systems. Patole et al. [296] used it with thymol in loaded chitosan dental film for the treatment of periodontitis, Esmaeili et al. [297] studied the effects of eugenol nanoemulsion as a topical delivery system, and Khayat et al. [298] developed eugenol microemulsion for transdermal delivery of drugs.

Aloe vera has great potential for biomedical applications because of its biological activity. For optimized properties, aloe vera has been added to chitosan in previous studies [299], but recently, researchers reported the production of aloe vera and bacterial cellulose composites [300,301]. No NC-based antimicrobial studies were detected in the literature reviewed.

Gupta et al. [302] investigated the antimicrobial activity of aloe vera loaded poly(vinyl alcohol) (PVA)/poly(ethylene oxide) (PEO)/carboxymethyl cellulose (CMC) coated polyester membranes. They evaluated antimicrobial properties against *S. aureus* and *E. coli*. The number of viable cells decreased in the membrane containing aloe vera as compared to a non-aloe vera control sample. aloe vera has been found to be effective against both Gram-positive and Gram-negative bacteria.

In future studies, it would be interesting to incorporate aloe vera into NC materials and to check their antimicrobial activity and efficacy. Laux and coworkers [303] claim that aloe vera gel and whole leaf could be used as an excipient in drug delivery systems. Their article is a review of the role of aloe vera leaf materials in oral, transdermal, and buccal drug delivery. Such materials could have good potential in various biomedical applications.

The beforementioned and some other studies of organic NC-based antimicrobial hybrids are summarized in Table 5.

## 4. Challenges and Future Prospects

NC extraction methods, raw material selection, environmental and biological toxicity, and life cycle assessment are extremely important for the development of sustainable applications in which NC has strong potential. NC can be extracted using different techniques from many different sources. In order to optimize the process and adapt the properties and characteristics of NC, it is important that future development include mainly sustainable, eco-friendly, and green isolation techniques, such as mechanical extractions and enzymatic hydrolysis looks the most promising. Most of the studies using advanced technology were successfully performed on a laboratory scale, and further research activities need to be conducted to transfer them to the industrial scale. Due to commercialization, it is necessary to look at the whole aspect and understand the impact on the environment that encompasses the life cycle of NC, as only this can enable its sustainable success. Consequently, the life cycle assessment of some environmental aspects of NC based materials should be done.

BNC is especially interesting, but unfortunately, due to shortcomings of scaling up BNC production to the industrial level, which is related to its current production costs due to the relatively expensive commonly used culture media and slow production processes, is this replacement currently less feasible.

In addition, not only wood biomass is of interest for obtaining NC, but also other plant biomass, which gives cleaner and morphologically/crystallinely different products, which are of particular interest in the pharmaceutical industry and biomedicine.

The development of materials science is also advancing rapidly in the biomedical field, so nanocellulosic materials may represent a promising solution in the future to overcome some of the insurmountable challenges of biomedical materials. Much of the emphasis in this review is on the use of NC in drug delivery. Manly, the physicochemical properties and biocompatibility of NC enable its usefulness for the controlled release of various model drugs in nearly all cases. The particularly encouraging is its compatibility with both hydrophilic, hydrophobic, water-soluble, and poorly water-soluble drugs. NC is rapidly experiencing its breakthrough into drug delivery systems, but further studies are required to completely address the environment and biological toxicity of NC-based materials (to microorganisms, animals, and humans). In that respect, the assessment of the potential risks attributed to the chemical modification of nanocellulosic material requires special attention.

Because many NC-based hybrids containing biopolymers, surfactants, bacteriocins, enzybiotics, phytophenols and other organic compounds have shown antimicrobial efficacy in the studies reviewed, the potential in this area is extremely high. Hence, the future lies mainly in natural extracts combined with NC (biomimetically structured materials), on which extremely little research has been done. Natural extracts, with their biocompatibility and nontoxicity, enable many applications. Given that natural extracts do not consist of only one component but contain several different ones, they can act synergistically. Thus, in vitro and in vivo studies are needed to predict the synergistic effect of natural extracts on organisms and to allow the breakthrough of such NC hybrids to actual use, especially in biomedicine.

Especially highlighting is also focused on the food industry, where the demand for new and innovative food packaging materials is growing. In particular, antimicrobial bionanocomposites are promising for the packaging of various problematic foods (e.g., meat products, dairy and bakery products, fruits and vegetables), where spoilage occurs due to microorganisms. A new packaging technology (active packaging) incorporates natural extracts into NC-based films to control microbial surface contamination of foods.

With the realization of the above-mentioned challenges, NC-based materials may certainly contribute to the improved quality of human life in the future, especially through the development of the next generation of NC hybrid materials.

## 5. Conclusions

Over the last decade, there has been an increasing number of research teams around the world who reported the use of nanocellulose/NC in drug delivery studies and modification of NC with different antimicrobial agents. This review aims to outline the current state of research and the future development of NC in antimicrobial and drug delivery applications through selected examples.

Most formulations of NC-based drugs can be used through various routes of administration and have demonstrated controlled and, in many cases, also sustained drug release due to the addition of NC. Moreover, NC shows tremendous potential for further study and even more successful use in controlled drug delivery systems.

The surface-modified antimicrobial films from NC present a wide range of possibilities in applications in the areas of medicine (e.g., wound healing), pharmacy (e.g., drug carriers), food packaging, etc., NC-based nanomaterials incorporated with both inorganic and organic antimicrobial agents showed extremely good antibacterial activity against both Gram-positive and Gram-negative bacteria. Some of the specifically functionalized NC materials also provide antifungal properties. In particular, NC materials with organic antimicrobials, because of their non-toxic and non-irritant properties, could be used as an alternative to ordinary inorganic antimicrobial agents with low molecular weight, whose bad features are potential toxicity and short-term antimicrobial efficacy. The mechanical properties of NC-based antimicrobial materials could be enhanced further. Above all, more environmentally friendly and biocompatible alternatives could be prepared since some natural materials provide good antimicrobial efficacy. NC-antimicrobial hybrids obtained from natural materials have great potential and could be used much more widely in biomedical applications.

Last but not least, NC-based antimicrobial tissues with incorporated nanoparticles can be very effective in fighting microorganisms and, consequently, infectious diseases. Overcoming antimicrobial resistance is necessary, as is the further development of new antimicrobial hybrids based on NC.

## Figures and Tables

**Table 1:** Diverse CNC-based delivery systems with specific modification agents, carrier forms, model drugs, and potential applications.

Co-Carrier	Modification Agent	Carrier Form	Model Drug	Possible Application	Ref.
Sodium alginate	CaCl_2_, EPTMAC, PEI	Hydrogel	Ceftazidime hydrate and epidermal growth factor human	Codelivery of drugs in the oral administration and wound dressing	[99]
Cyclodextrin	-	Complexes	Curcumin	Anticancer drug delivery systems	[100]
Quaternized cellulose	CHPTAC, β-GP	Hydrogel	Doxorubicin	Localized and sustained drug delivery depot systems for anticancer therapy	[101]
-	APTES, PPI-dendrimer, FA	Lyophilized NPs	Doxorubicin	Delivery of anticancer drug	[102]
-	Aln, ApA	Dispersion	--	Bone therapied and theranostics	[103]
Sodium alginate	-	Hydrogel	Ibuprofen	Drug carrier	[104]
PVA	-	Hydrogel lenses	Chitosan–poly(acrylic acid) NPs	Ophthalmic use as a drug carrier and as cornea regeneration implant	[105]
-	PVP	Film	Honey	Wound dressing for the treatment of chronic wounds	[106]
-	CTAB	Suspension	Curcumin	Drug carrier for hydrophobic drugs	[107]
-	PVA	Film	Curcumin	Antimicrobial drug delivery in a diabetic wound dressing	[108]
Chitosan	Tween 20, GA	Hydrogel	Curcumin	Drug delivery of curcumin	[110]
-	Tween 20	Solution	Curcumin	Delivery of curcumin to the stomach and upper intestinal tract	[111]
FO and PEI	-	Lyophilized hybrids	Doxorubicin	Layer-by-layer assembly with lysosomal pH-controlled drug release into the nucleus	[112]
PCL	-	Nanofibers	Tetracycline	Drug delivery system	[113]

CaCl_2_—calcium chloride, EPTMAC—2,3-epoxypropyl trimethylammonium chloride, PEI—polyethylenimine, CHPTAC—3-chloro-2-hydroxypropyltrimethylammonium chloride, β-GP—β-glycerophosphate, PPI-dendrimer—poly(propylene imine) dendrimer, APTES—(3-aminopropyl)triethoxysilane, FA—folic acid, Aln—sodium alendronate, ApA—3-aminoropylphosphoric acid, PVA—polyvinyl alcohol, PVP—polyvinylpyrrolidone, CTAB—cetyl trimethylammonium bromide, GA—glutaraldehyde, FO—folate, PCL—poly(ε-caprolactone).

**Table 2:** Diverse CNF-based delivery systems with specific modification agents, carrier forms, model drugs, and potential applications.

Co-Carrier	Modification Agent	Carrier Form	Model Drug	Possible Application	Ref.
-	LA	Dry foams and films	Riboflavin	Gastro retentive drug delivery	[115]
-	-	Nanopapers and nanofoams	Indomethacin	Fast and intermediate release profiles for drug delivery	[116]
-	-	Aerogels	Bendamustine hydrochloride	Carriers for oral drug delivery	[117]
HFBII	-	Emulsion	Naproxen and ibuprofen	Drug delivery applications via the oral route	[118]
-	GTMAC	Nanofoam	Furosemide	Prolonged drug delivery in the upper part of the gastrointestinal tract	[119]
Fe_3_O_4_ -Ag_2_O quantum dots	-	Powder	Etoposide and Methotrexate	Carrier of anticancer drugs for skin cancer	[120]
-	FITIC-DEX, lysozyme, and BSA	Hydrogel	Metronidazole, nadolol, and ketoprofen	Controlled delivery of several types of molecules	[121]
Sodium alginate	-	Beads (dried hydrogel)	Metformin hydrochloride	Drug carrier	[122]
Sodium alginate	-	Film	Ampicillin	Supporting material, drug delivery system for Tissue Engineering	[123]
PDA	calcium ion	Hydrogel	Tetracycline hydrochloride	Drug delivery vehicle	[124]
Chitosan	-	Film	Ketorolac tromethamine	Transdermal delivery systems	[125]
HPMC	-	Film	Ketorolac tromethamine	Food packaging and transdermal drug delivery applications	[126]
PLA	-	Hydrogel	Metoprolol and nadolol	Modulating sustained drug release	[127]
Gelatin	Dialdehyde starch	Cryogel	5-fluorouracil	Carrier for controlled drug release	[129]
Starch/pectin	Glycerin	Film	Methotrexate	Colonic drug release	[130]
-	-	Aerogel	Ibuprofen and amoxicillin	Drug delivery vehicle	[131]
-	-	Hydrogel	Ibuprofen	Controlled drug release system	[132]

LA—lauric acid sodium salt, HFBII—class II hydrophobin protein, GTMAC—glycidyltrimethylammonium chloride, FITIC-DEX—fluoresceinisothiocyanate-dextran, PDA—polydopamine, HPMC—hydroxypropylmethylcellulose, PLA—poly(lactic acid).

**Table 3:** Diverse BNC-based delivery systems with specific modification agents, carrier forms, model drugs, and potential applications.

Co-Carrier	Modification Agent	Carrier Form	Model Drug	Possible Application	Ref.
Starch/pectin	Glycerin	Film	Methotrexate	Colonic drug release	[130]
Poloxamer	-	Gel and micelle	Octenidine	Long-term dermal wound treatment and drug delivery	[133]
Collagen	-	Film	Lidocaine	Biodegradable microneedles for transdermal drug delivery	[134]
PMGly	MGly, AAPH, and MBA	Membranes	Diclofenac	Drug carriers for dermal and oral drug delivery	[135]
-	-	Hydrogel	BSA	Carrier for controlled delivery of proteins	[136]
-	-	Scaffolds	Caco-2-cells	Caco-2-based in vitro models of the human intestine	[137]
-	Glycerol	Membranes	Caffeine, ibuprofen, lidocaine and diclofenac	Topical drug delivery systems	[138]
-	-	Film	Crocin	Transdermal delivery of crocin	[139]

PMGly—poly(N-methacryloyl glycine), MGly—N-methacryloylglycine, AAPH—2,2′-azobis(2-methylpropionamidine) dihydrochloride, MBA—N,N′-methylenebis(acrylamide).

**Table 4:** Diverse inorganic NC-based antimicrobial hybrids.

Type of NC	Antimicrobial Agent/ Compound	Hybrid Form	Synthesis of Hybrids	Microbial Growth Inhibition	Ref.
BNC	AgNPs	Porous hybrids	Immobilization on the top and bottom surfaces of BNC by chemical interactions	*E. coli, S. aureus*	[23]
CNF	AgNPs	Coated papers	Impregnation of CNF with AgNPs’ solution	*E. coli, S. aureus*	[156]
BNC	AgNPs	Nanocomposites	Classical Tollens reaction and reduction of AgNO_3_ with NaBH_4_	*S. aureus*	[157]
CNC	ZnO	Films	Evaporation-induced self-assembly in aqueous solution	*E. coli, S. aureus*	[165]
	TiO_2_				
	Ag_2_O				
CNC/WG	TiO_2_	Films	TiO_2_ NPs were added to the WG/CNC suspension	*E. coli, S. cerevisiae, S. aureus*	[171]
CNF	TiO_2_	Nanocomposites	Cross-linking of TiNPs with BTCA and SHP	*E. coli, S. aureus*	[172]
CNF	CuO	Freeze-dried nanofibrils	“Green” reductive technique using an alcoholic extract of *Terminalia chebula fruit*	*C. albicans, E. coli, S. aureus*	[173]
CNC	ZnO	Nanocomposites	In situ solution casting technique	*E. coli, S. aureus*	[174]
CNF	ZnO	Paper sheets	Electrostatic assembly in aqueous medium using polyelectrolytes as macromolecular linkers	*B. cereus, K. pneumoniae, S. aureus*	[175]
BNC	ZnO	Films	Powdered BNC was dissolved in NMMO, and ZnO NPs were mixed into the BNC solution;	*E. coli*	[176]
BNC	ZnO	Sheets	Ultrasonic-assisted in situ synthesis	*E. coli, S. aureus*	[177]
BNC	MgO	Membranes	In situ: Sonochemical and wet chemical methods Ex situ: Immersion of BNC pellicles into a MgO dispersion	*E. coli, S. aureus*	[178]

AgNPs—silver nanoparticles, WG—Wheat gluten, TiNPs—titanium nanoparticles, BTCA—1,2,3,4-butanetetracarboxylic acid, SHP—sodium hypophosphite, CuNPs—copper nanoparticles, NMMO—N-methylmorpholine-N-oxide.

**Table 5:** Diverse organic NC-based antimicrobial hybrids.

Type of NC	Antimicrobial Agent (Additional Compound)	Hybrid Form	Synthesis of Hybrids	Microbial Growth Inhibition	Ref.
BNC	Benzalkonium chloride	Dry film	Immersion of BNC film into BZC solution	*B. subtilis, S. aureus*, *S. typhimurium*	[187]
BNC	Polyhexanide, povidone-iodine	Fleeces	Immersion of BNC samples in PI or PHMB	*S. aureus*	[188]
CNF from ginger	-	Film	Preparation of film with chemicals (alkalization, bleaching, acid hydrolysis) and ultrasonication	*B. subtilis, C. albicans*, *E. coli, P. aeruginosa*, *S. aureus*	[194]
CNF from ginger	-	Nanocomposites	Solvent-casting method reinforcement using PS and TS	*B. cereus*, *E. coli*, *S. aureus*, *S. typhimurium*	[304]
CNF from ginger	(Chitosan, PVA)	Nanocomposites	Solvent-casting method	*B. cereus*, *E. coli*, *S. aureus*, *S. typhimurium*	[305]
BNC	Chitosan	Hydrogel	Adding CS in culture media during bacterial cultivation	*E. coli*, *S. aureus*	[220]
NC	Chitosan	Nanocomposites	Mixing of chitosan solution, NC solution and glycerol solution	*E. coli*, *S. aureus*, *S. enteritidis*	[306]
CNC	Chitosan	Films	Flax CNC incorporated in CS film solution by following the solution casting method	*E. coli*, *E. faecalis*, *L. monocytogenes*, *P. aeruginosa*, *S. aureus*	[307]
CNC	Chitosan (PVP)	Film	Solution casting method	*P. aeruginosa*, *S. aureus*	[222]
CNC	Chitosan, PCL, grape seed extract	Film	Casting method for preparation of CS films with GSE and NC, the addition of PCL was achieved with coating and compression molding method for PCL	*E. coli*, *L. monocytogenes*	[234]
CNF	Chitosan, SNAP	Membrane	Encapsulation of SNAP in dispersed CS and mixed with CNFs	*E. faecalis*, *L. monocytogenes*, *S. aureus*	[231]
BNC	APS	Membrane	Surface functionalization with aminoalkyl groups	*E. coli*, *S. aureus*	[73]
BNC	Nisin, EDTA	Membrane	Immersion of BNC membranes into nisin solution with or without EDTA	*E. coli*, *S. aureus*	[243]
CNF	Nisin	Film	Immobilization of nisin on CNF using the coupling agent (EDC-NHS)	*B. subtilis*, *S. aureus*	[308]
CNC	Nisin (PLA)	Films	PLA-CNC films were treated with nisin by adsorption/diffusion coating method	*L. monocytogenes*	[309]
BNC	Bromelain	Membrane	Submersion of BNC into a BL solution	*E. coli*, *P. aeruginosa*, *S. aureus*	[249]
BNC	Laccase	Membrane	Physical enzyme immobilization: immersion of BNC into a laccase preparation	*E. coli*, *S. aureus*	[254]
CNC	Curcumin	Film	CNF suspended in PVA solution, and then curcumin was added	*B. coagulans*, *C. albicans*, *E. coli*, *P. mirabilis*, *S. aureus*, *Streptococcus* sp.	[108]
BNC	Freeze-dried curcumin	Membrane	Immersion method	*E. coli*, *S. aureus*	[310]
CNC	Carvacrol, curcumin (βCD, HPβCD)	Film	TOCNC-COONa and TOCNC-COOH were modified with βCD and HPβCD; Curcumin and carvacrol were entrapped by the attached HPβCD	*B. subtilis*	[311]
CNC	Rosin	Film	Esterification on CNC using rosin as the grafting agent and reaction solvent	*B. subtilis*, *E. coli*	[264]
CNF	Rosin, (PLA/chitosan)	Film	CNF modified by rosin by the SolReact process and then used as a reinforcement filler within the PLA matrix; the film was further coated with CS	*B. subtilis*, *E. coli*	[312]
CNF	Lysozyme	Aerogels	Physical immobilization of lysozyme: Mixing of the enzyme with CNFs followed by lyophilization	*E. coli*, *S. aureus*	[267]
BNC	Lysozyme	Dispersion	Physical absorption method to immobilize lysozyme onto BC nanofibers	*A. niger*, *E. coli*, *L. monocytogenes*, *S. aureus*, *S. cerevisiae*, *Y. enterocolitica*	[313]
CNC	Allicin	Nanocomposites	Modification of CNC with CA, further conjugation with allicin by a carbodiimide (EDC) cross-linker;	*A. niger, C. albicans*, *E. coli*, *S. aureus*	[274]
	Lysozyme		Coating of CNC with BSA and conjugated with lysozyme by the EDC method		
NC	Allicin	Fabric	Cellulose fabrics modified by APTES and conjugated with allicin-conjugated NC (EDC method)	*S. aureus*	[275]
CNC	Savory essential oil (agar)	Film	CNC suspension was dispersed in an AG solution; Tween 80 was added as the emulsifier, then SEO was added to the mixture	*B. cereus*, *E. coli*, *L. monocytogenes*, *S. aureus*	[276]

BZC—benzalkonium chloride, PHMB—polyhexanide, PI—Povidone-iodine, PS—potato starch, TS—tapioca starch, PVA—poly(vinyl alcohol), CS—chitosan, PVP—poly(vinyl pyrrolidone), PCL—polycaprolactone, GSE—grape seed extract, SNAP—S-nitroso-N-acetyl-d-penicillamine, APS—3-aminopropyltrimethoxysilane, EDTA—ethylenediaminetetraacetic acid, EDC—1-ethyl-3-(3-dimethylaminopropyl)carbodiimide, NHS—N-hydroxysuccinimide, PLA—poly(lactic acid), BL—bromelain, βCD—beta-cyclodextrin, HPβCD—hydroxypropyl-beta-cyclodextrin, TOCNC-COONa—oxidized cellulose nanocrystals with sodium carboxylate groups, TOCN-COOH—oxidized cellulose nanocrystals with free carboxyl groups; CA—citric acid, BSA—bovine serum albumin, APTES—aminopropyl triethoxysilane, AG—agar, SEO—savory essential oil.
